# Modeling the Cost Effectiveness of Neuroimaging-Based Treatment of Acute Wake-Up Stroke

**DOI:** 10.1371/journal.pone.0148106

**Published:** 2016-02-03

**Authors:** Ankur Pandya, Ashley A. Eggman, Hooman Kamel, Ajay Gupta, Bruce R. Schackman, Pina C. Sanelli

**Affiliations:** 1 Department of Health Policy and Management, Harvard School of Public Health, Boston, MA, United States of America; 2 Department of Healthcare Policy and Research, Weill Cornell Medical College, New York, NY, United States of America; 3 Department of Neurology, New York-Presbyterian/Weill Cornell Medical College, New York, NY, United States of America; 4 Department of Radiology, New York-Presbyterian/Weill Cornell Medical College, New York, NY, United States of America; 5 Department of Radiology, North Shore–LIJ Health System, Manhasset, NY, United States of America; Creatis UMR CNRS 5220, FRANCE

## Abstract

**Background:**

Thrombolytic treatment (tissue-type plasminogen activator [tPA]) is only recommended for acute ischemic stroke patients with stroke onset time <4.5 hours. tPA is not recommended when stroke onset time is unknown. Diffusion-weighted MRI (DWI) and fluid attenuated inversion recovery (FLAIR) MRI mismatch information has been found to approximate stroke onset time with some accuracy. Therefore, we developed a micro-simulation model to project health outcomes and costs of MRI-based treatment decisions versus no treatment for acute wake-up stroke patients.

**Methods and Findings:**

The model assigned simulated patients a true stroke onset time from a specified probability distribution. DWI-FLAIR mismatch estimated stroke onset <4.5 hours with sensitivity and specificity of 0.62 and 0.78, respectively. Modified Rankin Scale (mRS) scores reflected tPA treatment effectiveness accounting for patients’ true stroke onset time. Discounted lifetime costs and benefits (quality-adjusted life years [QALYs]) were projected for each strategy. Incremental cost-effectiveness ratios (ICERs) were calculated for the MRI-based strategy in base-case and sensitivity analyses. With no treatment, 45.1% of simulated patients experienced a good stroke outcome (mRS score 0–1). Under the MRI-based strategy, in which 17.0% of all patients received tPA despite stroke onset times >4.5 hours, 46.3% experienced a good stroke outcome. Lifetime discounted QALYs and costs were 5.312 and $88,247 for the no treatment strategy and 5.342 and $90,869 for the MRI-based strategy, resulting in an ICER of $88,000/QALY. Results were sensitive to variations in patient- and provider-specific factors such as sleep duration, hospital travel and door-to-needle times, as well as onset probability distribution, MRI specificity, and mRS utility values.

**Conclusions:**

Our model-based findings suggest that an MRI-based treatment strategy for this population could be cost-effective and quantifies the impact that patient- and provider-specific factors, such as sleep duration, hospital travel and door-to-needle times, could have on the optimal decision for wake-up stroke patients.

## Introduction

Time since stroke onset is a key clinical input in acute stroke treatment guidelines.[1] Thrombolytic treatment (i.e., tissue-type plasminogen activator [tPA]) is recommended for acute stroke patients with stroke onset time <4.5 hours, but not in patients with longer stroke onset times.[2] This information is unknown, however, in 14–28% of ischemic strokes, often because the stroke occurred during sleep (“wake-up” strokes).[3] Patients with unknown stroke onset time are generally excluded from thrombolytic treatments, although some studies have found that stroke severity is similar between wake-up and non-wake-up strokes.[4,5,6]

There has been interest in using neuroimaging information to identify wake-up stroke patients who would be eligible for tPA treatment. While both computed tomography (CT) and magnetic resonance imaging (MRI) modalities have been shown to predict early-onset stroke onset (either <3 or <4.5 hours) with some accuracy,[7,8,9,10] multi-sequence MRI has shown the most promise to guide treatment decisions.[3] Specifically, ischemic infarctions are detectable on diffusion-weighted imaging (DWI) within minutes on stroke onset, but these lesions take several hours to appear on fluid-attenuated inversion recovery (FLAIR); therefore, patients with mismatch infarcts (i.e., lesions appearing on DWI but not FLAIR) are more likely to have more recent time of onset of stroke.

A neuroimaging-based treatment decision rule has the potential to improve outcomes for wake-up stroke patients, but these benefits must be weighed against the treatment risks and healthcare costs associated with tPA treatment. The tradeoffs among these consequences will depend on the ability of the neuroimaging test to discriminate early-onset from late-onset wake-up strokes and other patient- and provider-specific factors. Therefore, the objective of this study was to evaluate the cost effectiveness of a neuroimaging-based decision rule for wake-up stroke patients compared to the status quo policy of no tPA treatment for these patients, and to identify patient- and provider-specific factors that could be used to guide individualized treatment decisions for this patient group.

## Methods

### Analytic Overview

We developed a computer-based model that simulates short- and long-term health and cost outcomes for wake-up stroke patients under different treatment strategies and model input assumptions. The simulated wake-up stroke patient population was 60% male with an average age of 65 years[11] and no contraindications to tPA treatment aside from stroke onset time. The model is comprised of three main parts: 1) a stroke onset module that assigns each patient a true stroke onset time during sleep and subsequent times from awakening to hospital arrival and treatment start; 2) an acute stroke health and cost module that determines short-term stroke mortality and morbidity effects based on stroke onset time and treatment status; and 3) a long-term health and cost module that projects post-stroke hospitalization health and cost estimates based on short-term stroke outcomes. We used the model to compare two treatment strategies upon arrival at the hospital: 1) completion of brain MRI with FLAIR and DWI sequences, followed by tPA administration for patients with “mismatch” infarcts and supportive care only for the remaining patients, or 2) no immediate MRI scans and supportive care for all patients. The model was programmed in TreeAge Pro 2014 (TreeAge Software Inc., Williamstown, Massachusetts).

### Stroke onset module and data

**Fig 1:**
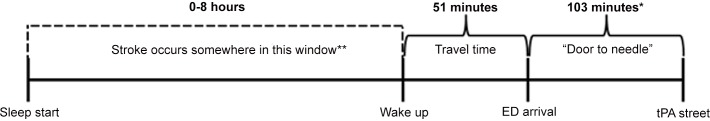
Time sequence from sleep start to tissue-type plasminogen activator (tPA) initiation for acute wakeup stroke patients in the micro-simulation model. *Door-to-needle time includes additional 30 minutes for performing and interpreting MRI. **Base-case assumption for true stroke onset during sleep is based on a uniform probability between 0–8 hours. Sleep durations of 4 and 6 hours, and alternative skewed beta distributions used in sensitivity analyses.

The stroke onset module starts when simulated patients go to sleep without any stroke symptoms. Fig 1 summarizes the steps used to calculate stroke onset time. The model uses a probability distribution, such as the uniform distribution (which was used in the base case) or beta distributions (which were used in sensitivity analyses, Figure A in S1 Appendix), to establish a true stroke onset time at some point between sleep start and wake up times. The duration of sleep was assumed to be eight hours in the base case, and was also varied in sensitivity analyses. After awakening, we estimated that it takes 51 minutes for a patient to become aware of the stroke and arrive at the hospital based on national registry data.[12] Upon hospital arrival, we estimated that it takes 103 minutes to start tPA treatment (“door-to-needle” time),[12] which included 30 minutes specifically for performing a limited brain MRI with DWI and FLAIR sequences at initial presentation.[13] Studies by Breuer et al. and Ebinger et al. suggest that door-to-needle times can be shorter (80 and 86 minutes, respectively) than 103 minutes (our base-case assumption), but these studies were conducted outside the U.S. and also had small sample sizes, which is why we conservatively chose to base our estimate on the large U.S.-based study by Fonarow et al.[12,14,15] Base-case model inputs and sensitivity analysis ranges are reported in Table 1.

**Table 1:** Model variables with base-case values and ranges used in one-way sensitivity analysis.

Variable	Base-case value	Sensitivity analysis range	Probability distribution for sensitivity analyses	Source(s)
*General model parameters*				
Age (years)	65	55–75	N/A	[11]
Male (%)	60	40–80	N/A	[11]
Discount rate (%)	3.0	N/A	N/A	Assumption
*Time-related variables*				
Sleep time (hours)	8	4, 6	N/A	Assumption
Time from wake up at home to hospital (min)	51	36–72 (IQR)	Gamma	[12]
Time from hospital arrival to tPA treatment start (“door-to-needle time”) (min)	77	60–98 (IQR)	Gamma	[12]
Additional time for MRI on presentation (min)	30	20–40	Gamma	Assumption; [13]
*Imaging diagnostic performance and tissue-type plasminogen activator (tPA) effectiveness*				
Sensitivity MRI to determine if stroke <4.5 hours	0.62	0.57–0.67	Beta	[9]
Specificity of MRI to determine if stroke <4.5 hours	0.78	0.72–0.84	Beta	[9]
Probability of mRS0-1 without tPA treatment	0.451	N/A	Dirichlet	[11]
Odds ratio of mRS0-1 with tPA treatment administered 0–180 minutes since stroke onset	1.75	1.35–2.27	Log normal	[16]
Odds ratio of mRS0-1 with tPA treatment administered 181–270 minutes since stroke onset	1.26	1.05–1.51	Log normal	[16]
Odds ratio of mRS0-1 with tPA treatment administered 271–360 minutes since stroke onset	1.00	0.95–1.40	Log normal	[16]
*Disease progression*				
Hazard ratio for non-stroke deaths for mRS0	1	N/A	Log normal	[24]
Hazard ratio for non-stroke deaths for mRS1	1	N/A	Log normal	[24]
Hazard ratio for non-stroke deaths for mRS2	1.11	1.0–1.3	Log normal	[24]
Hazard ratio for non-stroke deaths for mRS3	1.27	1.2–1.4	Log normal	[24]
Hazard ratio for non-stroke deaths for mRS4	1.71	1.3–2.0	Log normal	[24]
Hazard ratio for non-stroke deaths for mRS5	2.37	1.5–4.0	Log normal	[24]
Annual probability of recurrent stroke	0.051	0.02–0.065	Beta	[18,19]
Probability of death from recurrent stroke within 1 year	0.190	0.10–0.30	Beta	[18,19]
*Healthcare costs*				
Cost of MRI	488	390–586	Gamma	[21]
Cost of hospitalization for stroke without treatment	11,462	10,421–12,503	Gamma	[23,34]
Cost of hospitalization for stroke with treatment	18,182	16,798–19,565	Gamma	[23,34]
Annual cost post-hospitalization (mRS0-3)	5,293	4,234–6,351	Gamma	[22,34]
Annual cost post-hospitalization (mRS4-5)	13,557	10,846–16,268	Gamma	[22,34]
Cost of recurrent stroke hospitalization	20,079	16,063–24,095	Gamma	[19,43]
*Utilities*				
Utility of mRS0	0.8	0.8–1.0	Beta	[22,24,27,28]
Utility of mRS1	0.8	0.8–0.95	Beta	[22,24,27,28]
Utility of mRS2	0.65	0.68–0.9	Beta	[22,24,27,28]
Utility of mRS3	0.5	0.45–0.65	Beta	[22,24,27,28]
Utility of mRS4	0.35	0.1–0.4	Beta	[22,24,27,28]
Utility of mRS5	0.2	0.0–0.32	Beta	[22,24,27,28]

Note: All cost are reported in 2013 US dollars.

### Acute stroke module and data

In the acute stroke module, outcomes were based on modified Rankin Scale (mRS) scores, which range from 0 (no stroke symptoms) to 6 (death) with increasing severity (Figure B in S1 Appendix). Simulated wake-up stroke patients were assigned an mRS score based on mRS-specific outcome distributions for patients who did and did not receive tPA from the third European Cooperative Acute Stroke Trial (ECASS III)[11] (Table A in S1 Appendix). If patients were selected for treatment with tPA in the model, their probability of having a favorable stroke outcome (mRS 0–1) was modified based on time-specific tPA effectiveness values (odds ratio [OR] range of 1.75, 1.26, and 1.00 for stroke onset times of 0–180, 181–270, and 271–360 minutes, respectively) that were derived from a pooled analysis of nine large randomized tPA trials (including ECASS) by Emberson et al.[16] The increased risk of symptomatic intracranial hemorrhage (sICH) associated with tPA was not explicitly modeled, because the effects of sICH on stroke severity (and mortality) were already captured in the mRS outcome distribution; furthermore, the pooled analysis did not find a significant interaction between stroke age and tPA-related sICH events.[16]

### Post-stroke hospitalization module and data

Lifetime quality-adjusted life years (QALYs) and healthcare costs were simulated using a Markov state-transition model with mRS-based health states (Figure C in S1 Appendix). Patients entered the model with the mRS outcome from the acute stroke model. In yearly cycles patients were exposed to risks of dying from non-stroke causes[17] or having recurrent stroke (annual probability of 5.1%), 19% of which were fatal.[18,19] Patients who survived recurrent strokes were assigned to a higher (i.e., worse) mRS state with equal probability for all higher mRS states except death; the risk of acute recurrent stroke death, which is equivalent to an mRS score of 6, was already accounted for.[19]

### MRI-based decision rule

Although the model assigned each simulated patient a true stroke onset time, these times would be unknown to patients or providers because the strokes occurred during sleep. Using DWI-FLAIR mismatch from MRI would give providers information to decide whether wake-up stroke patients would be eligible for tPA based on onset time (<4.5 hours). This was operationalized in the model using sensitivity and specificity inputs for assessing hyper-acute (i.e., <4.5 hours) stroke onset time (where stroke onset time <4.5 hours is the affected state). Our base-case estimates for sensitivity (0.62) and specificity (0.78) came from a multi-center observational study in which two neuroradiologists assessed DWI-FLAIR mismatch for acute stroke patients with known onset times.[9] We performed extensive sensitivity analyses around these inputs because multiple studies have assessed the ability of neuroimaging information to discriminate early from late-onset strokes. [3,8]

### Costs and health-related quality-of-life

Costs associated with stroke and adverse events were estimated from a large national inpatient sample of community hospitals in the U.S. and from other published sources.[20,21,22,23] Costs of hospitalization for stroke with and without tPA treatment were $18,200 and $11,500 respectively, and included the costs of the intensive care unit, imaging, supplies, and nurse and administrator wages.[23] The MRI-based strategy included brain MRI costs.[21] Annual post-hospitalization costs varied depending on the patient’s mRS status and whether the patient experienced a recurrent (i.e., post-hospitalization) stroke events, which cost $20,000.[20,22] Utility weights representing health-related quality of life for extreme categories (i.e. mRS 0–1 and 5 categories) were based on survey data from a large sample of persons at increased risk for stroke using the time trade-off method to value hypothetical health states.[24] Utility weights for the remaining categories were assigned by linear interpolation, consistent with previously reported results.[25,26] Weights from other stroke-related utility studies were used in sensitivity analyses.[22,27,28]

### Model validation

We assessed internal model validity by replicating observed mRS outcomes from the ECASS III trial.[11,29] We assessed external validity by comparing model-generated results to observed acute stroke outcomes from sources that were not directly used to inform model inputs.[29,30] These outcomes and sources included: 1) the OR associated with tPA for mRS0-2 outcomes from a recent meta-analysis of 12 trials (including ECASS III) for acute stroke patients with known stroke onset time and stroke onset time between 0–3 hours;[31] and 2) the OR associated with tPA for patients with 1.5–3.0 hour stroke onset time (compared to 3.0–4.5 hours) for acute stroke death from a recent observational study.[32]

### Cost-effectiveness analysis

The QALYs and costs projected for each strategy were used to calculate an incremental cost-effectiveness ratio (ICER) for the image-based decision rule compared to no tPA treatment. This ICER was compared to a cost-effectiveness threshold of $100,000/QALY.[33] The analysis was conducted from a healthcare system perspective over a lifetime horizon, with all costs in 2013 dollars,[34] and future healthcare costs and QALYs were discounted at 3% annually.[35]

Sensitivity analyses were performed for alternative scenarios that assumed different sleep durations, times to treatment, MRI duration, and stroke onset distributions. Other model parameters were varied individually in one-way sensitivity analyses and a two-way sensitivity analysis was performed on the sensitivity and specificity of MRI to identify early- versus late-onset strokes. Overall model uncertainty was evaluated in probabilistic sensitivity analysis (PSA) by simultaneously conducting 10,000 random draws from pre-specified probability distributions for each variable (Table 1).[36]

## Results

Internal and external validation results are presented in Table B in S1 Appendix. Internal validation results indicate that the model was able to replicate the observed mRS outcomes for patients not receiving tPA treatment and to closely match mRS outcomes for patients receiving tPA treatment (differences in outcomes range from 0.007–0.009, i.e. 97%-101% of reported values). External validation results indicate that the ORs of favorable stroke outcome (mRS 0–2) for tPA versus no treatment were within the observed confidence interval from a recent meta-analysis for tPA treatment with stroke onset time <3 hours and stroke onset time >3 hours.

In the base case analysis, 23% of simulated patients had a stroke onset time <4.5 hours; the other 77% of patients had stroke onset time >4.5 hours. In the MRI-based strategy, this resulted in 17% of all patients receiving tPA with stroke onset time >4·5 hours due to the imperfect specificity of the MRI test. Overall, 46.3% of patients in the MRI-based strategy had a favorable stroke outcome (mRS0-1), compared to 45.1% of patients in the no treatment strategy. Assuming 20% of all ischemic strokes are wake-up strokes, and that the MRI-based strategy would result in 31% of these wake-up strokes being treated with tPA, and that 5.5% of ischemic strokes treated with tPA experience sICH, we estimated that the MRI-strategy would result in a 0.34 percentage point increase of sICH among ischemic stroke patients. As mentioned in the Methods section, the costs and health effects of these sICH events are already captured in the mRS results and costs of tPA treatment. Compared to the no treatment strategy, lifetime discounted QALYs were 0.4 higher and costs were $2,700 higher with the MRI-based treatment strategy, resulting in an ICER of $88,000/QALY (Table 2 and Table C in S1 Appendix).

**Table 2:** Lifetime per-person mRS0-1 outcomes, stroke onset time outcomes, inappropriately treated outcomes, quality-adjusted life years (QALYs), costs ($), and incremental cost-effectiveness ratios ($/QALY) for acute wakeup stroke patients.

Strategy	% mRS0-1	% <4·5 hours	% >4·5 hours	% inappropriately treated*	Life years	QALYs**	Costs**	ICER
8 hours sleep time								
No treatment	45.1	—	—	—	11.598	5.312	88,247	Reference
MRI-based strategy	46.3	23.0%	77.0%	16.9%	11.633	5.342	90,869	88,000
6 hours sleep time								
No treatment	45.1	—	—	—	11.598	5.312	88,247	Reference
MRI-based strategy	46.6	31.2%	68.8%	15.1%	11.645	5.355	91,082	66,000
4 hours sleep time								
No treatment	45.1	—	—	—	11.598	5.312	88,247	Reference
MRI-based strategy	47.2	45.9%	60.1%	11.9%	11.670	5.381	91,466	47,000

*defined as a percent of all patients that received tPA later than 270 minutes after stroke onset

**discounted at 3%

In scenarios where patients presented sooner after falling asleep than the full 8 hours modeled in the base case, the MRI-based strategy became more attractive (Table 2). As sleep time decreased (from 8 hours to 6 hours and 4 hours) the percent of patients with a stroke onset time <4.5 hours increased, the percent of patients in the MRI-based strategy with favorable stroke outcomes increased, and the percent in the MRI-based strategy treated with tPA despite a stroke onset time >4.5 hours decreased (to 15.1% and 11.9%, respectively). Decreases in sleep time also resulted in higher accumulated lifetime QALYS and costs for the MRI-based strategy compared to the no treatment strategy and therefore more favorable ICERs (e.g. for patients with 4 hours sleep duration, MRI-based strategy ICER <$50,000).

Model results were sensitive to scenarios that varied time to treatment and MRI duration inputs, with ICERs ranging from $47,000/QALY to $200,000/QALY under optimistic and pessimistic timing assumptions, respectively (Table D in S1 Appendix). The threshold value for time to hospital arrival was 55 minutes, which was fairly close to our base-case estimate of 51 minutes (Table E in S1 Appendix). In scenarios that varied the distributions for stroke onset during sleep times, the Beta (4,4) symmetrical distribution resulted in the no treatment strategy dominating the MRI-based strategy with only 5.6% of stroke onset times <4.5 hours. The Beta(3,5) distribution for a stroke onset time slightly skewed toward wake time resulted in 20.2% of true stroke onset times <4.5 hours (less than the base-case uniform distribution) and had an unfavorable ICER ($150,000/QALY) for the MRI-based strategy compared to no treatment (Table D in S1 Appendix). The two remaining stroke onset distributions, where stroke onset was more skewed towards wake time, resulted in ICERs <$100,000/QALY with 39.9%-87.9% of stroke onset times <4.5 hours.

**Fig 2:**
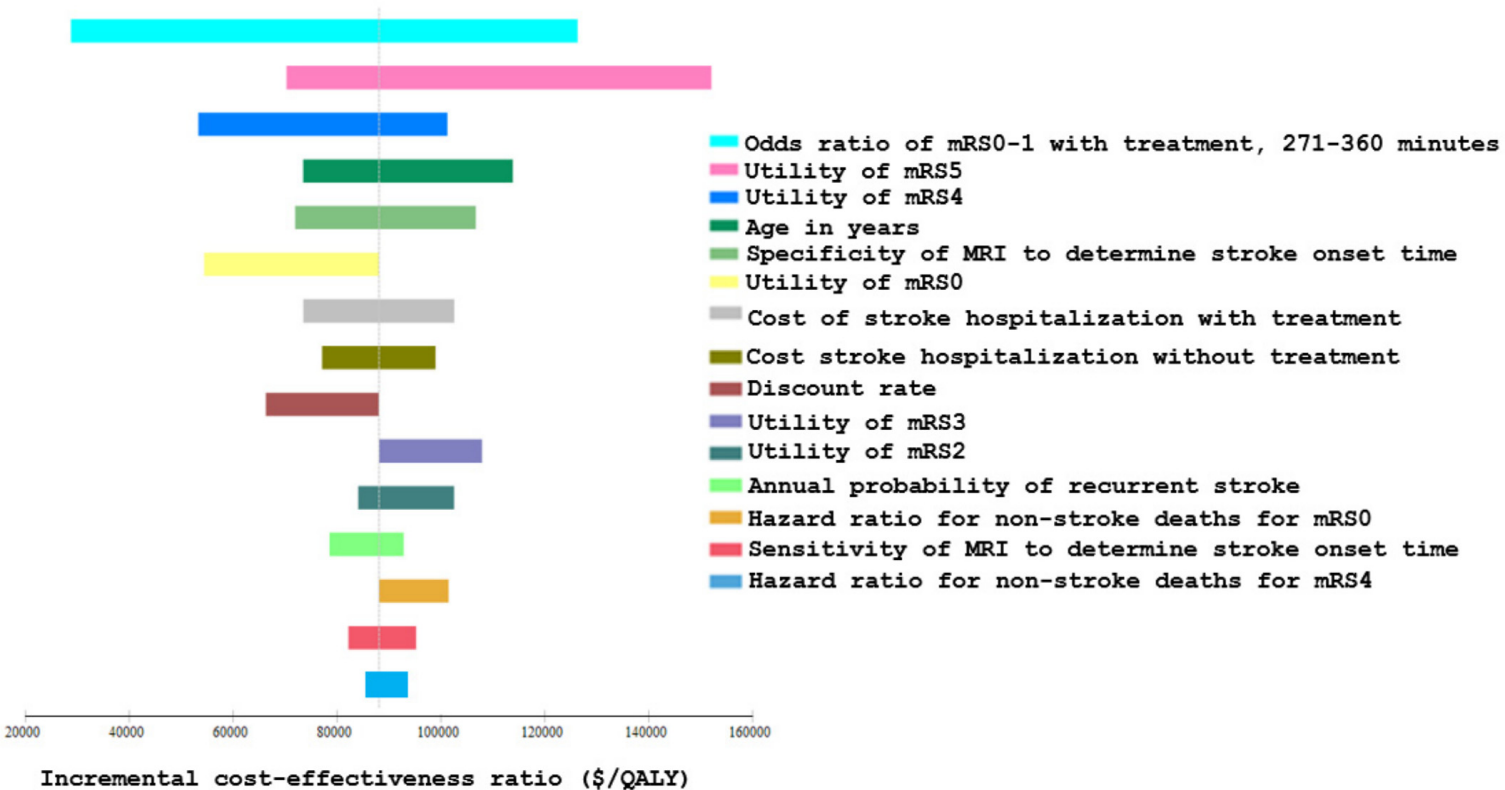
One-way sensitivity analysis results (ICERs for MRI-based strategy vs. no treatment). Tornado diagram summarizing one-way sensitivity analyses for the MRI-based strategy compared to the no treatment strategy for the base-case analysis. Most incremental cost-effectiveness ratios (ICERs) were close to the base-case result ($88,000/QALY) as model parameters were varied through plausible ranges, with the exceptions of the odds ratio of mRS0-1 with treatment for patients with stroke onset time 271–360 minutes, utility values for mRS5 and mRS4 health states, age in years, and MRI specificity. Parameters are shown in descending order of influence on model results.

**Fig 3:**
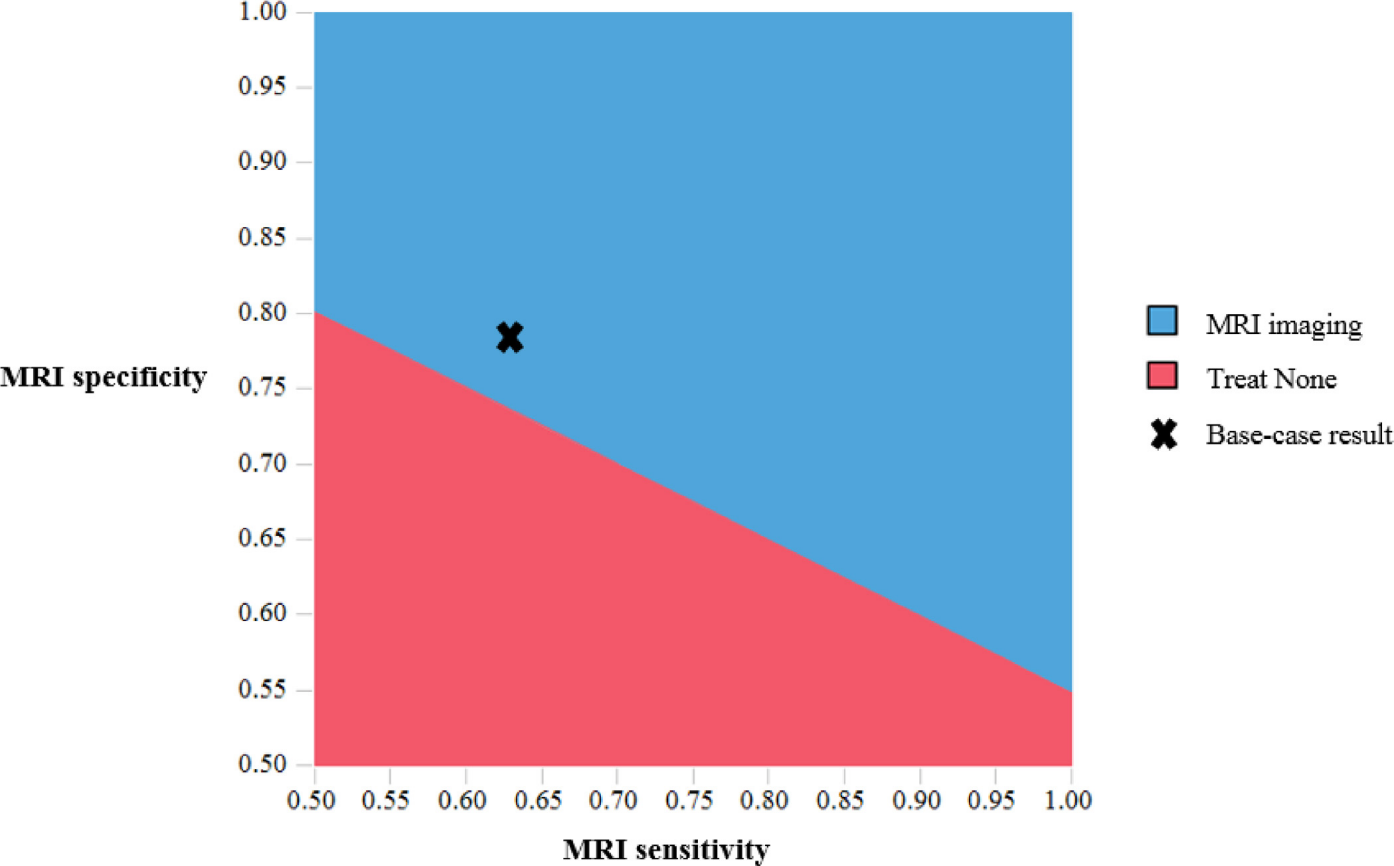
Two-way sensitivity analysis results for MRI sensitivity and specificity (for identifying stroke onset time of <4.5 hours), assuming willingness-to-pay for health of $100,000/QALY. The MRI-based strategy is optimal in the blue region, which includes the base-case result (marked by an “X”); the no treatment strategy is optimal in the red region given some other combinations of MRI sensitivity and specificity. The line separating the blue and red regions has an angle <45 degrees, which indicates that the results are more influenced by specificity than sensitivity.

**Fig 4:**
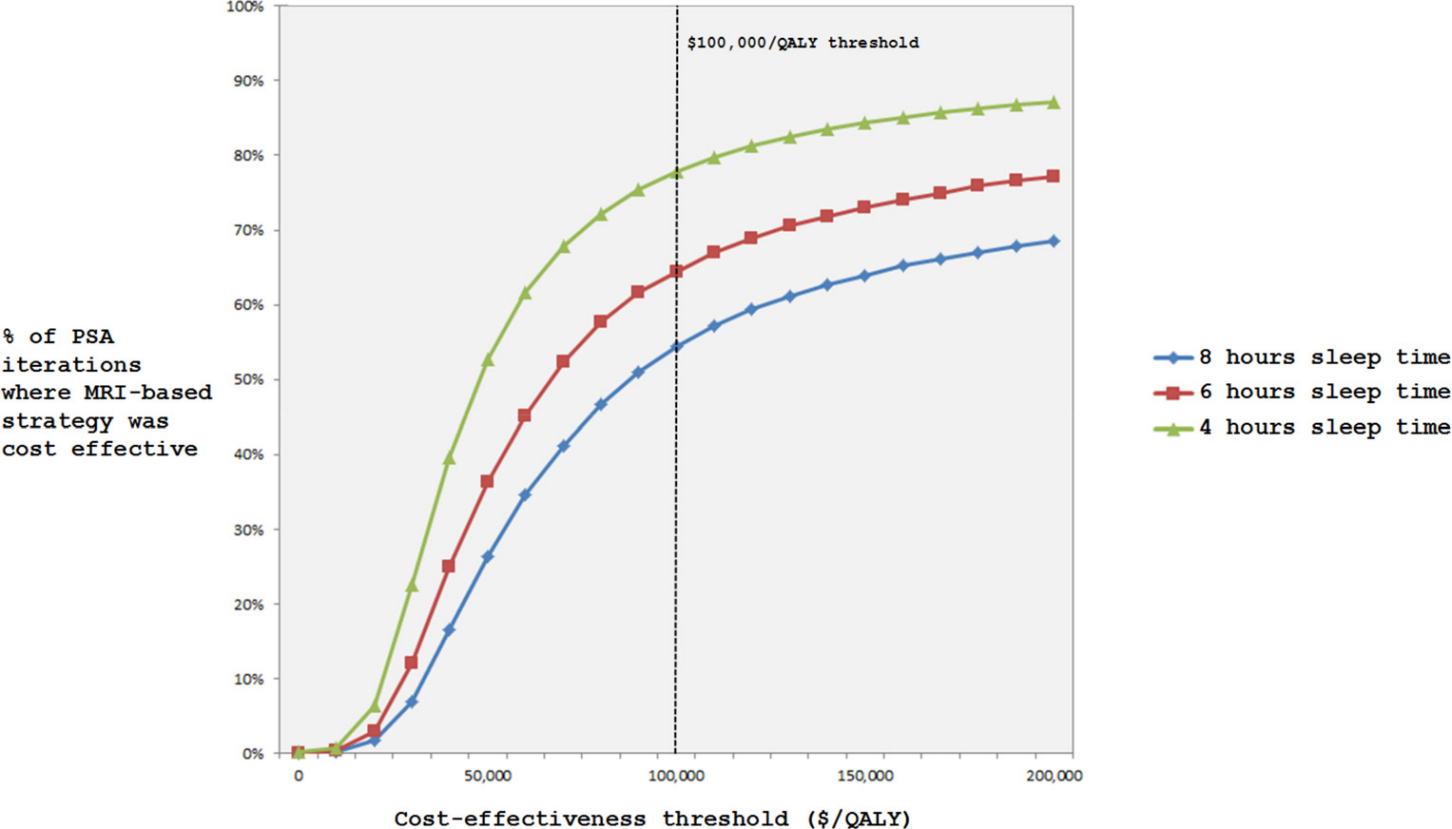
Cost-effectiveness acceptability curve from probabilistic sensitivity analysis. The probability of the MRI-based strategy and no treatment strategy being cost-effective plotted against the cost-effectiveness threshold. The probability of a strategy being cost-effective was based on the probabilistic sensitivity analysis, which incorporates the uncertainty of all model parameters.

Fig 2 shows ICER results for the fifteen most influential variables evaluated in one-way sensitivity analyses. ICER results were sensitive to uncertainty in the odds of a good outcome for those patients treated after 4.5 hours, utilities in the various outcome states, age and specificity of MRI to determine stroke onset time. Results were robust to plausible changes in sensitivity of MRI, acute and chronic stroke mortality parameters, and costs (Table E in S1 Appendix). Fig 3 presents the two-way sensitivity analyses results varying the sensitivity and specificity of MRI. It shows the ICER results are robust to plausible changes in MRI sensitivity but less robust to plausible changes in MRI specificity. For example, when MRI specificity decreases below 0.74 (holding sensitivity constant at 0.62), the no treatment strategy is optimal. In the PSA (Fig 4), the MRI-based strategy was optimal in 54%, 64%, and 78% of PSA iterations using a cost-effectiveness threshold of $100,000/QALY and 8, 6, and 4 hours of sleep time, respectively.

## Discussion

We developed an acute stroke simulation model for wake-up stroke patients and found that, using a cost-effectiveness threshold of $100,000/QALY, MRI-based treatment was a cost-effective option compared to the status quo policy of no treatment for these patients. Under base-case assumptions, the MRI-based strategy resulted in better short-term and long-term stroke outcomes, higher QALYs, and increased costs compared to the no treatment strategy resulting in a base-case ICER of $88,000/QALY. Our model performed well on internal and external validity checks compared to observed outcomes from large tPA studies.

The cost-effectiveness results were sensitive to several factors. ICERs for the MRI-based strategy were more favorable for wake-up stroke patients presenting sooner after falling asleep (after 4 or 6 hours compared to 8 hours of sleep). Sleep duration could be an important component of tailored treatment guidelines for wake-up stroke patients. Travel time to the hospital and door-to-needle times also affected the optimal treatment strategy, and could vary by patient and provider, and thus could be additional factors that guide individualized treatment policies. The diagnostic performance of using DWI-FLAIR mismatch was another model input that affected the ICER for the MRI-based strategy, particularly its ability to identify stroke ages >4.5 hours (i.e., MRI specificity). Future advances in neuroimaging techniques to improve the sensitivity and especially specificity of defining stroke onset would be valuable in the treatment of wake-up stroke patients.

Results were also influenced by the probability distribution used to model true stroke onset time, which highlights the importance of research on the prevalence of treatable wake-up strokes (i.e., onset times <4.5 hours). For instance, examining the prevalence of FLAIR-DWI mismatch in wake-up stroke patients can be used to approximate the true prevalence of true stroke onset time <4.5 hours. Additionally, estimating the proportion of wake-up strokes attributable to circadian rhythm-related blood pressure surges close to wake time could provide further insights into the true prevalence of stroke onset time <4.5 hours in wake-up stroke patients.[37]

Previous cost-effectiveness studies have shown that tPA is good value for money for patients with known stroke onset time.[19,22,38,39] Tung et al. reported an ICER of $23,000/QALY (in 2013 dollars) for tPA treatment in patients with known stroke age between 180–270 minutes. The ICER from our base-case analysis is somewhat higher ($88,000/year) because it reflects the overall effect of tPA treatment for some wake-up stroke patients with true stroke onset time <4.5 hours, which is cost-effective, and tPA treatment for some patients with true stroke onset time >4.5 hours, which negatively impacts health while increasing costs. Several small clinical trials have shown possible benefit from treating wake-up stroke patients and other larger going trials are testing tPA treatment for wake-up stroke patients;[3] our model compliments these efforts by highlighting the importance of key patient- and provider-specific variables (such as sleep duration, hospital travel and door-to-needle times) in base-case and “what-if” scenario analyses.

Our study has several limitations. First, simulation models require combining input sources from multiple sources. However, we feel confident in the model results based on our internal and external validation analyses. Second, we used sensitivity and specificity values for estimating stroke onset time greater than or less than 4.5 hours instead of category-specific likelihood ratios for >2 time categories (such as 0–90, 91–180, 181–270, and >270 minutes) because of relatively small sample sizes in the earlier time categories (0–90 and 91–181 minutes).[9] Third, true stroke onset during sleep influences the cost-effectiveness results from the model, but is also a parameter that is never truly known for wake-up stroke patients. We addressed this limitation by examining the effects of multiple stroke onset probability distributions and by conservatively assuming a uniform distribution in our base-case analysis. In previous studies, CT hypodensity findings were similar in wake-up stroke patients and patients with known stroke times, which suggests that wake-up strokes are more likely to occur close to wake time, which would imply that our findings are conservative with respect to the value of treating wake-up stroke patients.[3] Specifically, a study by Huisa et al. suggests that DWI-FLAIR mismatch was 43.7%, which is higher than we estimated with a uniform wake-up stroke distribution (23%), but the Huisa et al. study sample size was small (n = 22).[40]

## Conclusions

Wake-up stroke patients comprise 14%-28% of the patient population for a highly prevalent and important disease, but current treatment guidelines do not recommend tPA treatment for these individuals. Previous research suggests that brain imaging information could be used to identify wake-up stroke patients who might be eligible for tPA treatment, but clinical evidence is still being gathered. Specifically, there is not randomized controlled trial evidence for treating wake-up stroke patients with tPA,[41] but the WAKE-UP trial is currently being performed in Europe to address this question.[42] Our model-based study shows that an MRI-based treatment strategy for this population could be cost-effective and quantifies the impact that patient- and provider-specific factors, such as sleep duration, hospital travel and door-to-needle times, could have on the optimal decision for wake-up stroke patients. These findings could support the rationale for future clinical trials of individualized wake-up stroke treatment guidelines.

## Supporting Information

S1 Appendix Includes figures and tables that provide more information about the model structure and results. (DOCX)

S1 Dataset Data file used to generate probabilistic model results. (XLS)
