# Supplementary material for: Modeling the Cost Effectiveness of Neuroimaging-Based Treatment of Acute Wake-Up Stroke
Source: PLoS One. 2016 Feb 3;11(2):e0148106. doi: 10.1371/journal.pone.0148106 (PMC4740488; doi:10.1371/journal.pone.0148106)

**Online Supplements**

Table A. mRS state distributions after acute stroke for placebo and tissue-type plasminogen activator (tPA) before adjusting treatment effects with time since stroke onset

| **Health state** | **No tPA** | **tPA** | **Probability distribution for sensitivity analyses** | **Source** |
| --- | --- | --- | --- | --- |
| mRS 0 | 0.218 | 0.275 | Dirichlet | ^11^ |
| mRS 1 | 0.233 | 0.249 | Dirichlet | ^11^ |
| mRS 2 | 0.164 | 0.141 | Dirichlet | ^11^ |
| mRS 3 | 0.114 | 0.093 | Dirichlet | ^11^ |
| mRS 4 | 0.137 | 0.093 | Dirichlet | ^11^ |
| mRS 5 | 0.052 | 0.081 | Dirichlet | ^11^ |
| mRS 6 | 0.082 | 0.067 | Dirichlet | ^11^ |

Table B. Internal and external model validation results

| **Acute Stroke Outcome** | **Validation type** | **Model result** | **Target value and/or range** | **Source for target** | **Adjustments made to model for validation analysis*** |
| --- | --- | --- | --- | --- | --- |
| mRS0-2, no tPA (<3 hours onset) | Internal | 0.615 | 0.615 | ^11^ | 100% of onset times between 1.5-3.0 hours |
| mRS0-2, tPA (<3 hours onset) | Internal | 0.656 | 0.665 | ^11^ | Same as above |
| mRS4-6, no tPA (<3 hours onset) | Internal | 0.271 | 0.271 | ^11^ | Same as above |
| mRS4-6, tPA (<3 hours onset) | Internal | 0.248 | 0.241 | ^11^ | Same as above |
| OR of mRS0-2 for tPA (<3 hours onset) vs. no treatment | External | 1.56 | 1.53 (1.26-1.86) | ^29^ | Onset times between 0-3 hours; favorable stroke outcome defined as mRS0-2 (instead of mRS0-1) |
| OR of mRS0-2 for tPA (>3 hours onset) vs. no treatment | External | 1.11 | 1.07 (0.96-1.20) | ^29^ | Same as above |
| OR of mRS6 for tPA at 1.5-3.0 hours vs. tPA at 3.0-4.5 hours | External | 0.82 | 0.86 (0.78-0.96) | ^30^ | All onset times either 1.5-3.0 hours or 3.0-4.5 hours |

*Stroke onset times were assumed to be known and were adapted to recreate source data conditions for model validation runs

Table C. Intermediate outcome and cost effectiveness results from decision analytic model using base-case input parameter values

| **Strategy** | **%mRS0-1** | **%mRS4-6** | **Life years** | **Life years*** | **QALYs** | **QALYs*** | **tPA costs*** | **Non-tPA costs*** | **Total costs*** | **ICER** |
| --- | --- | --- | --- | --- | --- | --- | --- | --- | --- | --- |
| 8 hours sleep time | | | | | | | | | | |
| No treatment | 45.1 | 54.9 | 11.598 | 9.114 | 6.706 | 5.312 | $0 | $88,247 | $88,247 | Reference |
| MRI-based strategy | 46.3 | 53.7 | 11.633 | 9.140 | 6.746 | 5.342 | $5,680 | $85,189 | $90,869 | $88,000 |
| 6 hours sleep time | | | | | | | | | | |
| No treatment | 45.1 | 54.9 | 11.598 | 9.114 | 6.706 | 5.312 | $0 | $88,247 | $88,247 | Reference |
| MRI-based strategy | 46.6 | 53.4 | 11.645 | 9.150 | 6.763 | 5.355 | $6,270 | $84,812 | $91,082 | $66,000 |
| 4 hours sleep time | | | | | | | | | | |
| No treatment | 45.1 | 54.9 | 11.598 | 9.114 | 6.706 | 5.312 | $0 | $88,247 | $88,247 | Reference |
| MRI-based strategy | 47.2 | 52.8 | 11.670 | 9.167 | 6.796 | 5.381 | $7,338 | $84,128 | $91,466 | $47,000 |

*discounted at 3%

Table D. Scenario analysis cost-effectiveness results varying treatment start times, MRI times and stroke onset distributions

| **Strategy** | **% mRS0-1** | **% <4·5 hours** | **% >4·5 hours** | **% inappropriately treated*** | **Life years** | **QALYs**** | **Costs**** | **ICER** |
| --- | --- | --- | --- | --- | --- | --- | --- | --- |
| 90 min to treatment; MRI 45 min | | | | | | | | |
| No treatment | 45.5 | -- | -- | -- | 11.598 | 5.312 | 88,247 | Reference |
| MRI-based strategy | 46.1 | 17.8% | 82.2% | 18.1% | 11.617 | 5.325 | 90,735 | 200,000 |
| 90 min to treatment; MRI 30 min | | | | | | | | |
| No treatment | 45.5 | -- | -- | -- | 11.598 | 5.312 | 88,247 | Reference |
| MRI-based strategy | 46.4 | 21.0% | 79.0% | 17.4% | 11.625 | 5.333 | 90,818 | 120,000 |
| 90 min to treatment; MRI 20 min | | | | | | | | |
| No treatment | 45.5 | -- | -- | -- | 11.598 | 5.312 | 88,247 | Reference |
| MRI-based strategy | 46.5 | 23.0% | 77.0% | 16.9% | 11.631 | 5.340 | 90,868 | 94,000 |
| 60 min to treatment; MRI 45 min | | | | | | | | |
| No treatment | 45.5 | -- | -- | -- | 11.598 | 5.312 | 88,247 | Reference |
| MRI-based strategy | 46.3 | 23.8% | 76.2% | 16.8% | 11.634 | 5.344 | 90,887 | 84,000 |
| 60 min to treatment; MRI 30 min | | | | | | | | |
| No treatment | 45.5 | -- | -- | -- | 11.598 | 5.312 | 88,247 | Reference |
| MRI-based strategy | 46.8 | 26.9% | 73.1% | 16.1% | 11.644 | 5.354 | 90,963 | 65,000 |
| 60 min to treatment; MRI 20 min | | | | | | | | |
| No treatment | 45.5 | -- | -- | -- | 11.598 | 5.312 | 88,247 | Reference |
| MRI-based strategy | 47.0 | 29.1% | 70.9% | 15.6% | 11.649 | 5.361 | 91,021 | 57,000 |
| 45 min to treatment; MRI 45 min | | | | | | | | |
| No treatment | 45.5 | -- | -- | -- | 11.598 | 5.312 | 88,247 | Reference |
| MRI-based strategy | 46.8 | 26.9% | 73.1% | 16.1% | 11.644 | 5.354 | 90,964 | 65,000 |
| 45 min to treatment; MRI 30 min | | | | | | | | |
| No treatment | 45.5 | -- | -- | -- | 11.598 | 5.317 | 88,247 | Reference |
| MRI-based strategy | 47.0 | 30.1% | 69.9% | 15.4% | 11.653 | 5.364 | 91,044 | 54,000 |
| 45 min to treatment; MRI 20 min | | | | | | | | |
| No treatment | 45.5 | -- | -- | -- | 11.598 | 5.312 | 88,247 | Reference |
| MRI-based strategy | 47.3 | 32.5% | 67.5% | 14.9% | 11.662 | 5.373 | 91,097 | 47,000 |
| Stroke Onset Base-case: Uniform(0,1) | | | | | | | | |
| No treatment | 45.1 | -- | -- | -- | 11.598 | 5.312 | 88,247 | Reference |
| MRI-based strategy | 46.3 | 23.0% | 77.0% | 16.9% | 11.633 | 5.342 | 90,869 | 88,000 |
| Stroke Onset Right-skewed: Beta(3,5) | | | | | | | | |
| No treatment | 45.1 | -- | -- | -- | 11.598 | 5.312 | 88,247 | Reference |
| MRI-based strategy | 45.9 | 20.2% | 79.8% | 17.6% | 11.621 | 5.329 | 90,800 | 150,000 |
| Stroke Onset Right-skewed: Beta(2,5) | | | | | | | | |
| No treatment | 45.1 | -- | -- | -- | 11.598 | 5.312 | 88,247 | Reference |
| MRI-based strategy | 46.8 | 39.9% | 60.1% | 13.2% | 11.654 | 5.364 | 91,392 | 61,000 |
| Stroke Onset Right-skewed: Beta(1,8) | | | | | | | | |
| No treatment | 45.1 | -- | -- | -- | 11.598 | 5.312 | 88,247 | Reference |
| MRI-based strategy | 50.0 | 87.9% | 12.1% | 2.7% | 11.767 | 5.483 | 90,542 | 25,000 |
| Stroke Onset Bell-shape: Beta(4,4) | | | | | | | | |
| No treatment | 45.1 | -- | -- | -- | 11.598 | 5.312 | 88,247 | Reference |
| MRI-based strategy | 45.4 | 5.6% | 94.4% | 20.8% | 11.601 | 5.309 | 90,411 | DOMINATED |

*defined as a percent of all patients that received tPA later than 270 minutes after stroke onset

**discounted at 3%

*Note: All scenarios assume 8 hours of sleep*

Table E. Deterministic threshold analysis results given cost-effectiveness threshold of $100,000/QALY

| **Variable** | **Base-case value** | **Image-based strategy optimal** | **Pre-specified sensitivity values or range** |
| --- | --- | --- | --- |
| Age (years) | 65 | < 71 years | 55-75 |
| Male (%) | 60.0 | All values | 40-80 |
| Maximum sleep time (hours) | 8 | All values | 4-8 |
| Time from wake up at home to hospital (min) | 51 | < 55 min | 36-72 (IQR) |
| Time from hospital arrival to tPA treatment start (“door-to-needle time”) (min) | 77 | < 81 min | 60-98 (IQR) |
| Additional time for MRI on presentation (min) | 30 | < 36 min | 20-40 |
| Sensitivity MRI to determine if stroke <270 minutes | 0.62 | All values | 0.57-0.67 |
| Specificity of MRI to determine if stroke <270 minutes | 0.78 | > 0.73 | 0.72-0.84 |
| Odds ratio of mRS0-1 with tPA treatment administered 0-180 minutes since stroke onset | 1.75 | > 1.62 | 1.35-2.27 |
| Odds ratio of mRS0-1 with tPA treatment administered 181-270 minutes since stroke onset | 1.26 | > 1.23 | 1.05-1.51 |
| Odds ratio of mRS0-1 with tPA treatment administered 271-360 minutes since stroke onset | 1.00 | > 0.99 | 0.95-1.40 |
| Hazard ratio for non-stroke deaths for mRS2 | 1.11 | All values | 1.0-1.3 |
| Hazard ratio for non-stroke deaths for mRS3 | 1.27 | All values | 1.2-1.4 |
| Hazard ratio for non-stroke deaths for mRS4 | 1.71 | All values | 1.3-2.0 |
| Hazard ratio for non-stroke deaths for mRS5 | 2.37 | All values | 1.5-4.0 |
| Annual probability of recurrent stroke | 0.051 | All values | 0.02-0.065 |
| Probability of death from recurrent stroke within 1 year | 0.190 | All values | 0.10-0.30 |
| Cost of MRI | 488 | All values | 390-586 |
| Cost of hospitalization for stroke without treatment | 11,462 | All values | 10,421-12,503 |
| Cost of hospitalization for stroke with treatment | 18,182 | < 19,300 | 16,798-19,565 |
| Annual cost post-hospitalization (mRS0-3) | 5,293 | All values | 4,234-6,351 |
| Annual cost post-hospitalization (mRS4-5) | 13,557 | All values | 10,846-16,268 |
| Cost of recurrent stroke hospitalization | 20,079 | All values | 16,063-24,095 |
| Utility of mRS0 | 0.80 | All values | 0.80-1.0 |
| Utility of mRS1 | 0.80 | All values | 0.80-0.95 |
| Utility of mRS2 | 0.65 | < 0.81 | 0.68-0.90 |
| Utility of mRS3 | 0.50 | < 0.62 | 0.45-0.65 |
| Utility of mRS4 | 0.35 | < 0.40 | 0.10-0.40 |
| Utility of mRS5 | 0.20 | > 0.14 | 0.00-0.32 |

Figure A. Beta distributions used to determine stroke onset during sleep

Panel A: Right-skewed; Beta(3,5)


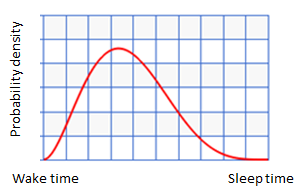


Panel B: Right-skewed; Beta(2,5)


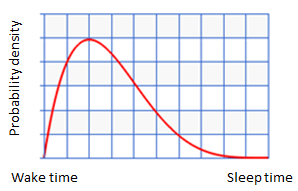


Panel C: Right-skewed; Beta(1,8)


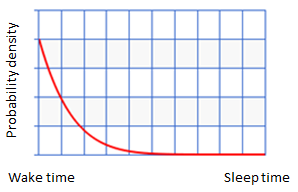


Panel D: Bell-shape; Beta(4,4)


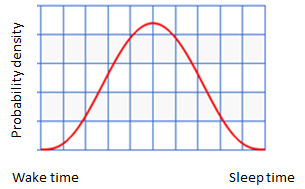


*Note: Probability density is defined here as the relative likelihood for stroke onset to take place on a given time during sleep. Sleep duration times used were 8, 6, and 4 hours in separately analyses.*

Figure B. Simplified depiction of acute stroke module


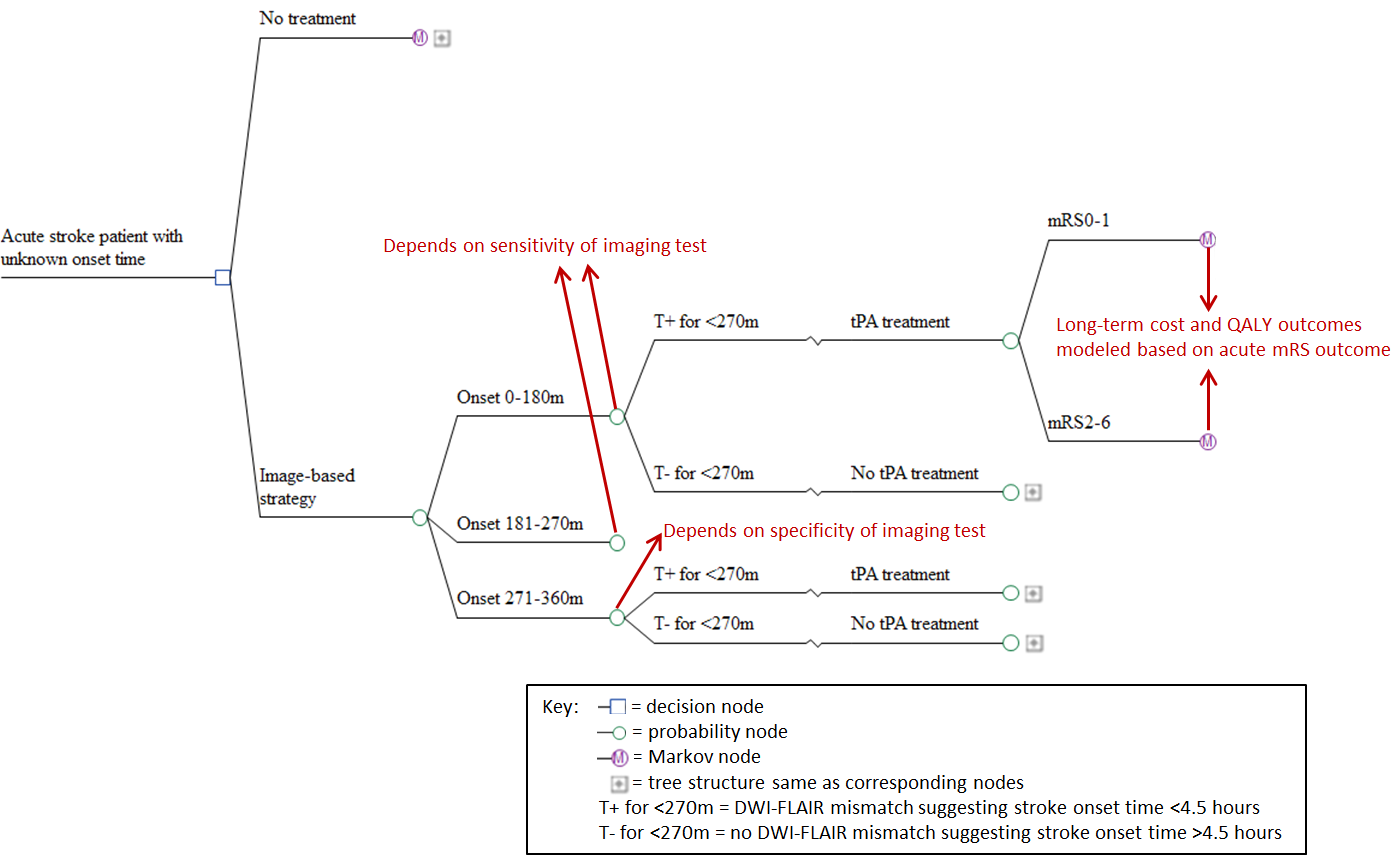


Figure C. Simplified depiction of post-stroke hospitalization module


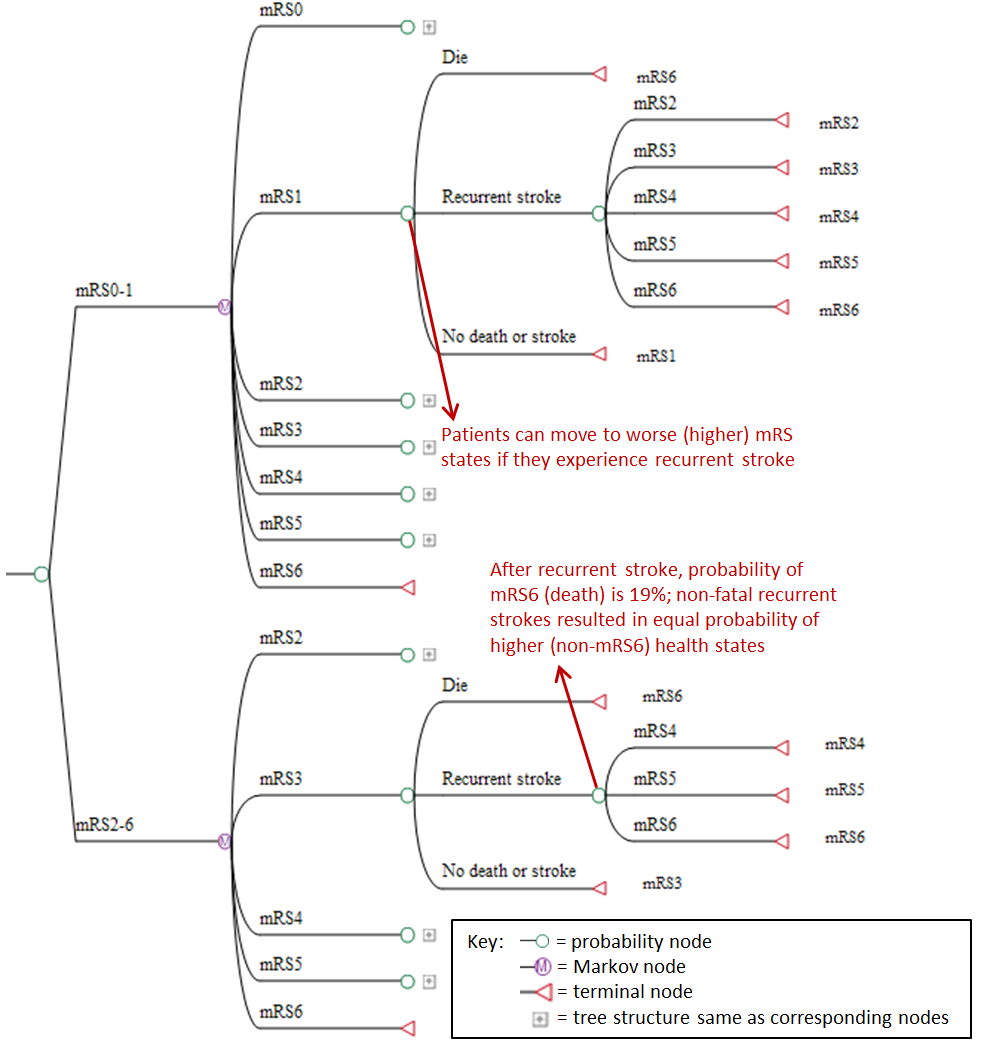

Supplement: S1 Appendix — (DOCX) [file pone.0148106.s001.docx]
